# Gene delivery to the hypoglossal motor system: preclinical studies and translational potential

**DOI:** 10.1038/s41434-021-00225-1

**Published:** 2021-02-11

**Authors:** Brendan M. Doyle, Michele L. Singer, Thomaz Fleury-Curado, Sabhya Rana, Ethan S. Benevides, Barry J. Byrne, Vsevolod Y. Polotsky, David D. Fuller

**Affiliations:** 1 Department of Physical Therapy, University of Florida, Gainesville, FL USA; 2 McKnight Brain Institute, University of Florida, Gainesville, FL USA; 3 Rehabilitation Science PhD Program, University of Florida, Gainesville, FL USA; 4 Breathing Research and Therapeutics Center, University of Florida, Gainesville, FL USA; 5 Department of Pediatrics and Powell Gene Therapy Center, University of Florida, Gainesville, FL USA; 6 Division of Pulmonary and Critical Care Medicine, Department of Medicine, Johns Hopkins University School of Medicine, Baltimore, MD USA

**Keywords:** Diseases, Neuroscience

## Abstract

Dysfunction and/or reduced activity in the tongue muscles contributes to conditions such as dysphagia, dysarthria, and sleep disordered breathing. Current treatments are often inadequate, and the tongue is a readily accessible target for therapeutic gene delivery. In this regard, gene therapy specifically targeting the tongue motor system offers two general strategies for treating lingual disorders. First, correcting tongue myofiber and/or hypoglossal (XII) motoneuron pathology in genetic neuromuscular disorders may be readily achieved by intralingual delivery of viral vectors. The retrograde movement of viral vectors such as adeno-associated virus (AAV) enables targeted distribution to XII motoneurons via intralingual viral delivery. Second, conditions with impaired or reduced tongue muscle activation can potentially be treated using viral-driven chemo- or optogenetic approaches to activate or inhibit XII motoneurons and/or tongue myofibers. Further considerations that are highly relevant to lingual gene therapy include (1) the diversity of the motoneurons which control the tongue, (2) the patterns of XII nerve branching, and (3) the complexity of tongue muscle anatomy and biomechanics. Preclinical studies show considerable promise for lingual directed gene therapy in neuromuscular disease, but the potential of such approaches is largely untapped.

## Introduction

The lingual or hypoglossal (XII) motor system is comprised of the intrinsic and extrinsic muscles of the tongue and the motoneurons which innervate them. Dysfunction and/or reduced output of the XII motor system can contribute to conditions such as dysphagia, dysarthria, and sleep disordered breathing. The fundamental thesis of this article is that gene therapy targeting the XII motor system has therapeutic potential for a variety of lingual-related disorders, but this potential is largely untapped. Gene therapy targeting the XII motor system offers two general strategies for treating lingual disorders. First, correcting tongue and/or XII motoneuron pathology in genetic neuromuscular disorders may be readily achieved by lingual directed delivery of viral vectors. The retrograde movement of vectors such as adeno-associated virus (AAV) enables targeted gene delivery to XII motoneurons following lingual delivery. Second, conditions with impaired or reduced tongue muscle activation can potentially be treated using viral-driven chemo- or optogenetic approaches to activate XII motoneurons and/or tongue myofibers.

### Tongue muscles and motoneurons

The tongue is comprised of eight skeletal muscles that perform mechanically complex movements and cause three-dimensional changes in tongue shape [1]. The complex lingual muscle architecture makes it difficult to precisely identify the biomechanical impact of individual muscles, but lingual muscles work in concert to execute tongue movements during behaviors such as eating, breathing, and oral communication [2].

The four intrinsic tongue muscles consist of myofibers completely contained within the body of the tongue and their contraction alters tongue shape. The complexity of the intrinsic musculature is highlighted by the concept of the tongue as a “muscular hydrostat”. In this regard, the tongue can be thought of as a cylindrical structure with a constant volume, and small decreases in diameter can produce significant increases in length [3]. The four extrinsic tongue muscles originate from bony attachment sites or connective tissues of the hyoid bone, mandible, and styloid process [4–6]. Extrinsic muscle contraction can protrude or retract the tongue as well as depress the tongue base. Table 1 summarizes the innervation and mechanical actions of the tongue muscles based on prior publications [7–10].

**Table 1:** Innervation and mechanical actions of the intrinsic and extrinsic tongue muscles.

Muscle	Motoneurons	Nerve	Mechanical action
Genioglossus *(Extrinsic)*	Lateral and centrolateral aspects of the ventral compartment	XII, Medial branch	Tongue protrusion (posterior fibers), tongue retraction (anterior fibers), and depress tongue body
Hyoglossus *(Extrinsic)*	Lateral aspect of the dorsal compartment	XII, Lateral branch	Depresses and retracts tongue body, elevates hyoid bone
Palatoglossus *(Extrinsic)*	Nucleus ambiguus	X, Pharyngeal branch	Posterior tongue elevation, grooves the tongue, and depresses soft palate
Styloglossus *(Extrinsic)*	Lateral aspect of the dorsal compartment	XII, Lateral branch	Elevation and retraction of tongue
Inferior longitudinal *(Intrinsic)*	Dorsomedial aspect of the dorsal compartment	XII, Medial and lateral branches	Depress tongue tip, shortens tongue (retraction)
Superior longitudinal *(Intrinsic)*	Dorsomedial aspect of the dorsal compartment	XII, Lateral branch	Elevates tongue tip, shortens tongue (retraction)
Transversus *(Intrinsic)*	Medial aspect of ventral compartment	XII, Medial branch	Narrows and elongates tongue (protrusion)
Verticalis *(Intrinsic)*	Medial aspect of ventral compartment	XII, Medial branch	Flattens and elongates tongue (protrusion)

Tongue innervation via the XII nerve undergoes extensive branching as the main trunk approaches the body of the tongue [4, 11, 12]. Humans and rodents have a distinct medial XII branch which innervates the primary protrusor muscle, the genioglossus [7]. The primary retractor muscles, the hyo- and styloglossus, are innervated by a lateral XII branch with variable presentation in humans [7]. The medial and lateral XII branches could be particularly useful in the context of targeted gene delivery studies to protrusor or retractor muscles.

The XII motor nucleus is a somatotopically organized, longitudinal column in the dorsal medulla. Generally, XII motoneurons located dorsally innervate retractor muscles, whereas ventrally located motoneurons innervate protrusors [6]. The ventral nucleus is further subdivided with medial cells innervating intrinsic protrusor muscles and lateral cells innervating extrinsic protrusors [13]. Within the dorsal nucleus, cells innervating intrinsic retractor muscles are located dorsomedially, and cells projecting to extrinsic retractors are located laterally [14]. The somatotopic segregation of XII motoneurons into functional groups also appears to extend to their biophysical properties. A recent investigation found that motoneurons of intrinsic tongue muscles had greater resting membrane potentials (i.e., more depolarized) as compared to genioglossus motoneurons [15].

### Tongue motor impairments

Coordinated tongue movements enable manipulation and posterior transport of a food bolus. Accordingly, dysphagia (disordered swallow function) is often present when lingual function is compromised [16–18]. Consequences include aspiration, pneumonia, airway obstruction, dehydration, weight loss, and malnutrition [19, 20]. Motor speech impairments such as dysarthria have been associated with diminished strength, endurance, and/or motor activation of lingual muscles [21–23]. The consequences of dysarthria extend beyond impaired communication and can considerably reduce overall quality of life [24–26]. Therapeutic approaches to address lingual deficits include diet modification, compensatory postural changes and/or environmental adjustments [27, 28]. Other treatments include mechanical or thermal stimulation, oromotor strengthening exercises, and electrical stimulation [25, 29–31]. These treatments represent strategies to help improve or compensate for lingual motor dysfunction, but they do not address the underlying pathology. In this regard, gene therapy may represent a novel therapeutic option, but there has been limited work on this topic [32].

Obstructive sleep apnea (OSA) is present in 10% of the population and lingual mechanisms figure prominently in the etiology. Specifically, sleep-related reductions of tongue muscle activation contribute to or cause upper airway narrowing or collapse. Of the tongue muscles, the genioglossus has been particularly emphasized, beginning with the seminal work of Remmers who hypothesized that reduced activity during sleep led to airway obstruction [33]. Decreases in genioglossus muscle activity are normal during sleep, but are greater in OSA patients [34]. Mechanistically, decreases in tongue muscle activity during sleep appear to reflect decreased noradrenergic input to XII motoneurons during NREM [35] and increased muscarinic cholinergic input during REM [36].

Treatments for OSA include surgical intervention and chronic positive airway pressure. In addition, since decreased tongue muscle activity leads to OSA, much research has focused on stimulating XII motor output during sleep. Direct stimulation of the XII nerve [37] can decrease pharyngeal collapsibility [38], relieves airflow obstruction [39], and can decrease OSA severity [40]. Pharmacological treatments are limited, but clinical trials targeting the noradrenergic and cholinergic systems show promise [41–43] and may suggest targets for gene therapy. Desipramine, a norepinephrine reuptake inhibitor, can reduce OSA severity in some patients [43]. The combination of atomoxetine (a norepinephrine reuptake inhibitor) and oxybutynin (an antimuscarinic drug) is more effective [42]. Thus, treatments that increase tongue muscle activation can be effective in treating OSA. Initial work in animal models (see next section) suggests that gene therapy strategies to excite XII motoneurons may also be effective [44].

### Targeted gene delivery to the XII motor system using AAV

**Fig. 1:**
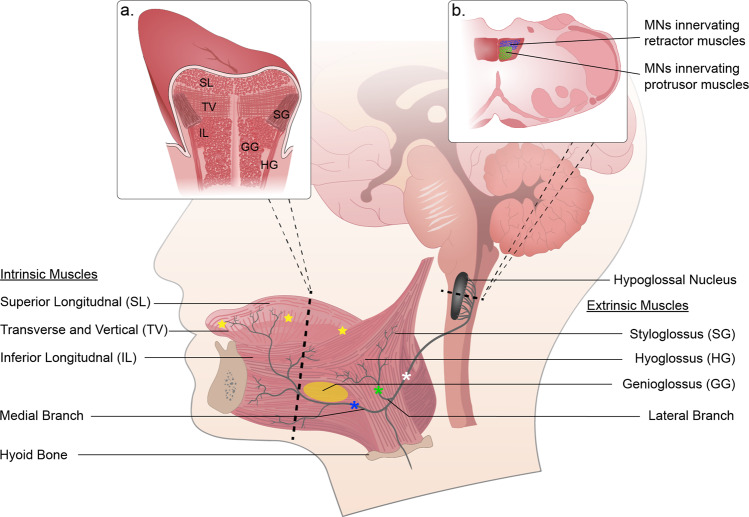
Schematic diagram of the hypoglossal (XII) motor system emphasizing potential sites for viral injection. The XII motor nucleus innervates the tongue via cranial nerve XII. The intrinsic muscles form the body of the tongue and the extrinsic muscles insert into the body of the tongue. The main XII nerve trunk bifurcates with the lateral branch innervating extrinsic retractors and the medial branch innervating extrinsic protrusors. Both branches also innervate intrinsic tongue muscles. The star symbols show potential injection sites for targeted gene delivery directly to the main body of the tongue. The yellow oval on the genioglossus represents a potential site of viral injection if the goal is selective activation of this muscle (or associated XII motoneurons) using opto- or chemogenetics. Potential sites for direct XII nerve injection include the medial branch (blue asterisk), lateral branch (green asterisk) and main trunk (white asterisk). Direct stereotaxic viral delivery to the XII nucleus is also possible in preclinical studies. Inset panels: **a** coronal section of the tongue illustrating organization of extrinsic and intrinsic muscles; **b** horizontal section of the medulla highlighting somatotopic organization of XII motoneurons.

The accessibility of the tongue (Fig. 1) and foundational knowledge of its innervation and basic biomechanics (Table 1) make it amenable to targeted gene delivery using viral vectors. Thus, gene delivery to the tongue and XII motoneurons has utility in preclinical studies of respiratory and/or lingual motor control, as well as potential for gene therapy for lingual-related disorders. In regard to therapeutic applications, we suggest two primary strategies. First, in cases of genetic neuromuscular disorders with lingual pathology, targeted viral delivery to the tongue and/or XII motoneurons may “correct” pathology and improve XII motor function. Second, in cases where activation of the tongue muscles is inadequate, direct excitation of tongue myofibers and/or XII motoneurons could be achieved using viral-driven opto- or chemogenetic strategies. Both approaches require efficient delivery of viral vectors to the XII motor system, and this is reviewed next.

### Targeting the tongue via intralingual viral delivery

Intralingual vector delivery effectively drives gene and protein expression in the tongue [32, 45–47] and can reverse tongue muscle histopathology in neuromuscular disease [45–47]. Pompe disease is a neuromuscular disorder associated with mutations in a single gene (acid alpha glucosidase or GAA), macroglossia, dysphagia, and dysarthria [48]. Thus, a murine Pompe model (*Gaa*^*−/*−^) was used to determine if intralingual AAV injections could produce sustained GAA expression with reversal of the tongue myofiber histopathology [45]. A single injection of AAV9-DES-GAA to the tongue base was highly effective in driving transgene (GAA) expression localized to the posterior tongue. Tissues harvested 4 months after intralingual AAV9 therapy showed robust GAA expression with concomitant reductions in glycogen accumulation. The AAV injection was insufficient to drive transgene expression across the anterior–posterior length of the tongue, but this could be circumvented through multiple injections targeting anterior and posterior regions.

Viral vectors can also drive gene expression in lingual taste cells via direct injection into the tongue submucosa [49, 50]. Several serotypes of AAV have been screened for this purpose including AAV1, 2, 5, 6, as well as a lentiviral vector; however, none were effective at driving gene expression in taste cells. However, a synthetic AAV serotype known as AAV-DJ was highly effective at driving gene expression in all functional taste cell types [49, 50]. The AAV-DJ has a hybrid capsid created from 8 AAV serotypes via DNA family shuffling technology, and demonstrates higher infectivity rates across a broad range of cells and tissue types [51]. Gene expression was also observed in nongustatory epithelial cells as well as the underlying mesenchymal and lingual muscle cells. These studies illustrate that beyond targeting lingual motor disorders, tongue gene transfer may have utility in elucidating the roles of specific proteins in taste cell development and gustation [49, 50].

The local (e.g., site of intramuscular injection) and/or systemic immune response is always a potential concern with viral gene transfer [52]. The host immune response can reduce or even negate the treatment through targeting of viral particles and/or elimination of cells expressing the transgene. In our studies of single injections of AAV9 and AAV2 to the murine tongue [45, 46, 53] we have not observed a significant local immune response (i.e., at or near the site of AAV delivery). Further, following tongue injection with AAV9, persistent XII motoneuron transgene expression was observed at the longest time point we evaluated (1 year) following the AAV delivery [46]; longer time intervals have not been assessed in the lingual system. Attention to the immune response is of particular importance when translating AAV therapies from preclinical animal models to human application. Antibodies to AAV can be found in many humans due to environmental exposure to AAV [54], and the murine immune response to AAV is different than in humans [55]. Nevertheless, transgene expression has been confirmed up to 5 years following intramuscular AAV injection in humans [56], thereby demonstrating the potential of this approach for long-term correction of physiological deficits. Immune management is a major area of emphasis in clinical trials of AAV, and the reader is directed to recent reviews of this topic [57, 58]

The immune response will be a particular concern if multiple AAV injections are separated in time, and should be carefully evaluated and monitored in any therapeutic study of AAV delivery [57, 59]. However, if multiple intralingual viral injections are delivered during the same acute surgical session (e.g., to better infect the entire tongue), this should circumvent the immune response that would likely accompany AAV injections separated by days to weeks.

Another challenge of lingual viral injections targeting specific tongue muscles is the complex architecture of the tongue (Fig. 1). This makes focal delivery to a particular intrinsic or extrinsic tongue muscle technically difficult, such as targeting the primary tongue protrusor the genioglossus. Targeting a specific lingual muscle is not likely to be a concern in therapeutic studies of lingual muscle function in neuromuscular disorders such as Pompe disease [45], but will be a concern in studies aimed at delivering light or chemically-activated proteins to selective muscles (see next section). Relative specificity of tongue muscle transduction is possible in smaller species with low injection volumes [44], and the accuracy of lingual viral injections should improve as the size of the tongue increases, making it easier to delivery small injection volumes to specific muscles.

### Targeting XII motoneurons using intralingual AAV delivery

Direct skeletal muscle injection of viral vectors followed by retrograde axonal transport provides a means to target gene delivery directly to a specific motoneuron pool. Retrograde targeting of motoneurons by AAV was first shown by Kasper et al. [60] and multiple AAV serotypes, including 1, 2, 5, 6, 7, 8, and 9 can show retrograde movement in select neural circuits [60–66]. Several studies confirm that intralingual delivery of AAV (serotypes 1 and 9) can drive long-term gene expression in XII motoneurons [45–47, 53], and targeting genioglossus motoneurons can be achieved with reasonable specificity [44]. The first report was from Elmallah et al. who delivered AAV9-CBA-GFP to the base of the tongue in adult mice. Expression of GFP was present at 8 weeks post-injection in an average of 234 ± 43 XII motoneurons [53]. The AAV9 results were compared to a highly efficient retrograde tracer (cholera toxin-β) which labeled approximately fourfold more XII neurons. The total number of XII motoneurons in an adult mouse is between 900 and 1600 [67, 68], and thus the AAV9 method infected between 15 and 25% of the motoneuron pool. This is consistent with a subsequent report indicating that intralingual AAV9 can infect 32% of the XII motoneuron pool [44]. Direct comparisons of AAV1 vs. AAV9 indicate that AAV9 is considerably more effective at retrograde movement following intralingual delivery [46]. More recent work raises the possibility of XII motoneuron “cross correction” via lingual AAV9 delivery [45]. Pompe (*Gaa*^−^^*/−*^) mice received an intralingual injection of AAV9 which encoded a modified GAA protein designed for improved cellular trafficking. The modified GAA was detected in ~200% more XII motoneurons as compared to treatment with AAV9 encoding a “normal” GAA protein. Cross correction could occur if GAA secreted from one AAV9 transduced XII motoneuron moves via receptor mediated uptake into adjacent motoneurons and is subsequently trafficked to lysosomes.

Intralingual AAV therapy also shows promise in treating amyotrophic lateral sclerosis (ALS). In the SOD1^G93A^ mouse model of ALS, a single intralingual treatment with AAV driving expression of microRNA targeting the superoxide dismutase 1 (SOD1) gene (AAVrh10-H1-miR^SOD1^) dramatically reduced SOD1 mRNA expression in the tongue, and caused a small (~1 wk) but statistically significant increase in lifespan in females [32]. The lingual treatment also improved aspects of breathing function but did not impact swallow as studied using videofluoroscopy. Another study in the same ALS mouse model found that lingual treatment with AAVrh10-H1-miR^SOD1^ preserved the integrity of tongue neuromuscular junctions, but did not impact survival of XII motoneurons [47].

**Fig. 2:**
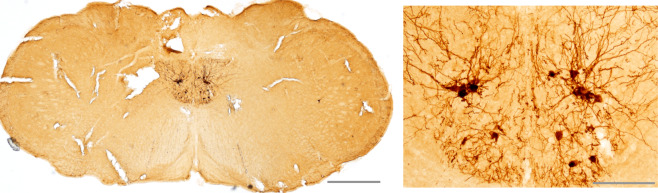
Bilateral XII motoneuron transgene expression following intralingual injection of AAV9-GFP. The right panel shows a higher power view of both XII nuclei. XII motoneuron staining can be seen to extend beyond the soma and with extensive axonal/dendritic branching. Scale bars: left panel, 500 µm; right panel, 100 µm.

As an example, Fig. 2 shows XII motoneuron gene expression following direct intralingual AAV9 delivery. This example is from our laboratory using adult mice and shows robust XII motoneuron transgene expression 12 weeks following delivery of 20 µl of AAV9-CBA-GFP to the base of the tongue. To our knowledge, there is no evidence that the virus first infects muscle cells before moving across the neuromuscular junction. Rather, viral transport is a multistep process that begins with binding of the capsid to a cell surface receptor [69]. The initial uptake requires cell surface glycan receptors that vary by serotype [70, 71]. Once in the cell, AAV9 is first trafficked into nonmotile endosomes, then exocytic vesicles and a retrograde-directed late endosome/lysosome compartment [72]. Endosomal transport is driven by cytoplasmic dynein and requires Rab7 function [72]. Similar mechanisms occur in serotypes AAV1 and AAV8 [66].

Several methods could potentially increase the relative number of transduced XII motoneurons following tongue delivery of viral vectors. For example, myelin is a potential barrier for uptake of the AAV capsid into the nerve terminal, and transient demyelination via ethidium bromide can increase retrograde transduction efficiency up to sixfold in large diameter sensory neurons [73]. Demyelinating XII motor axons, even transiently, would likely dramatically impair tongue motor control. More practical strategies may be to utilize multiple AAV injections and/or provide larger amounts of viral particles [73–76], or to utilize a more efficient retrograde vector. For example, a new rAAV variant (rAAV2-retro) has been created by directed evolution to increase the propensity for retrograde transport. While AAV2 and AAV9 showed similar labelling efficiency as rAAV2-retro at the site of injection, rAAV2-retro can enhance retrograde labelling by up to two orders of magnitude, at least in corticopontine projections [77]. The degree of labelling of rAAV2-retro rivals that of synthetic retrograde tracers within most neural systems tested and is consistently superior to other AAV serotypes [77].

### Targeting XII motoneurons via viral delivery directly to the XII nerve

**Fig. 3:**
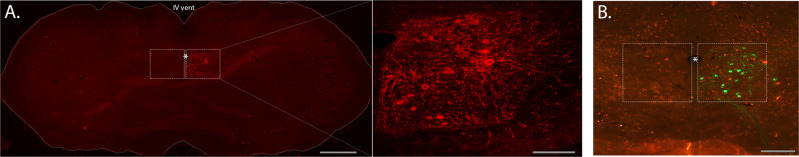
Motoneuron transgene expression following direct delivery of AAV9-GFP to the XII nerve. **A** shows GFP expression visualized using a fluorescent secondary antibody. **B** GFP fluorescence in XII motoneuron soma. In both panels, the boxes show the approximate locations of the left and right XII nucleus and the * indicates the central canal. The images confirm XII motoneuron transgene expression is present ipsilateral to the AAV9 injection to the XII nerve. IV vent = fourth ventricle. Scale bars: **A** left panel, 500 µm; right panel, 100 µm. **B** 250 µm.

Since uptake of AAV requires binding to cell surface receptors [70, 71], direct delivery into the XII nerve using a needle or pipette may allow for increased uptake into axons. This was tested in a study of sciatic nerve vs. gastrocnemius delivery of AAV2 in mice, with nerve delivery infecting a greater proportion of motoneurons [78]. This has not been directly tested in the XII motor system, but on theoretical grounds direct nerve injection should increase efficacy. Proof-of-concept data shown in Fig. 3 illustrate that direct XII nerve injection with AAV9 can produce robust XII motoneuron transgene expression. Another consideration regarding nerve injection is that it should require considerably lower volumes as compared to intramuscular delivery [78], and this could lessen immune reactions. Following intramuscular AAV injections, transgene expression can be limited by immune-related abolition of viral particles, and/or cells expressing the transgene [79]. Particular challenges related to direct nerve delivery include the complexity of the XII nerve branching (e.g., Fig. 1) and the possibility for damage to the nerve due to the injection method. Nevertheless, an experienced surgeon will likely be able to isolate and microinject the main trunk of the XII nerve, or the prominent medial and lateral branches.

### Targeting XII motoneurons using stereotaxic brainstem delivery

Delivery of gene transfer vectors into the medulla via stereotaxic injection is the most direct way to target XII motoneuron transduction [80–82]. For example, Horton et al. injected AAV8 to the XII nucleus using stereotaxic coordinates and histologically verified highly localized transgene expression in the XII nucleus [80]. Similar findings have been reported using AAV5 [81, 82]. Brainstem injections are unlikely to be clinically translatable due to surgical challenges and risks associated with inserting a needle into the medulla, but this approach enables proof-of-concept preclinical studies. One limitation of direct intraparenchymal brainstem viral delivery is that it will be virtually impossible to uniquely target a specific muscle group (e.g., only genioglossus motoneurons), although it should be possible to target regions of the XII motor nucleus, such as dorsal vs. ventral.

### Tongue activation using chemogenetic and optogenetic approaches

Designer Receptors Exclusively Activated by Designer Drugs (DREADD) technology has been utilized to directly manipulate XII motor output in at least four published studies [44, 80–82]. This approach uses selectively engineered muscarinic acetylcholine receptors that have been altered so that they no longer bind native ligands, but instead can be activated by select small molecules that are otherwise physiologically inert [83]. G-protein associated DREADDs can be utilized to stimulate, inhibit or silence the activity of cell populations [83]. Commonly, DREADD mediated excitation of neural circuits is achieved by expressing Gq signaling coupled muscarinic hM3Dq receptors in target neurons. These neurons can then be activated by systemic administration clozapine-N-oxide (CNO) [83] or related compounds [84] which act as ligands. Alternatively, neuronal inhibition can be achieved by expressing Gi signaling coupled muscarinic hM4Di receptors.

The first two descriptions of how expressing DREADDs in the XII nucleus could be used to activate the tongue were published almost simultaneously in 2017 [80, 81]. Fleury-Curado et al. delivered an excitatory DREADD construct (rAAV5-hSyn-hM3(Gq)-mCherry) or a control virus (rAAV5-hSyn-EGFP) directly to the XII motor nucleus using stereotaxic surgery in C57BL/6J mice. Approximately 2 months later, delivery of the CNO ligand induced an increase in genioglossus muscle EMG activity accompanied by dilation of the pharyngeal airway as assessed using MRI. Horton et al. used a ChAT-Cre mouse model coupled with stereotaxic delivery of a cre-dependent vector (AAV8-hSyn-DIO-hM3Dq-mCherry) to the XII motor nucleus. At 4 weeks post-AAV injection, CNO caused a sustained increase in tongue muscle EMG, but diaphragm EMG output was unaffected. The sustained increase in tongue motor activity was present during both non-REM and REM sleep. Together, these two seminal reports demonstrated that chemogenetics can be used to directly stimulate XII motoneurons with functional impact [80, 81].

A subsequent publication demonstrated that DREADDs can be used to inhibit XII motoneurons and thereby cause sleep disordered breathing [82]. An inhibitory construct was packaged in AAV5 (AAV5-hM4Di-mCherry) and delivered to the XII nucleus of C57BL/6J mice via direct stereotaxic injection. One month later, CNO administration caused airway obstruction during both REM and non-REM sleep. This work was supplemented with in vitro neurophysiology studies of brainstem slices in which direct intracellular recording confirmed that CNO caused a decrease in XII motoneuron bursting. A more recent publication has advanced the field by taking advantage of the capability for AAV9 to move retrogradely to target XII motoneurons following tongue injection [44]. Obese mice that display frequent upper airway obstruction during sleep were treated with an intralingual injection of AAV9 encoding an excitatory DREADD construct (AAV9-hSyn-hM3(Gq)-mCherry). Histological studies confirmed that the virus effectively drove transgene expression in the XII motor nucleus, primarily in the ventromedial region containing genioglossus motoneurons. At 6–8 weeks following tongue injection, intraperitoneal delivery of a DREADD ligand caused a sixfold increase in genioglossus activity with concomitant increases in pharyngeal airway patency and increased airflow during non-REM sleep. DREADD-induced increases in genioglossus activity were not unique to inspiration, but rather occurred across the entire respiratory cycle. Two aspects of this work are particularly important for potential translation of chemogenetics in the context of sleep disordered breathing. First, the virus was delivered using direct tongue injection. This simple procedure [85] effectively transduced a sufficient number of XII motoneurons to enable a beneficial physiological response. Second, a novel DREADD ligand, JHU37160 [84] was used. The more commonly used CNO ligand has substantial off target effects, but JHU37160 can be delivered at much lower concentrations and has reduced risk of off target effects [84].

**Fig. 4:**
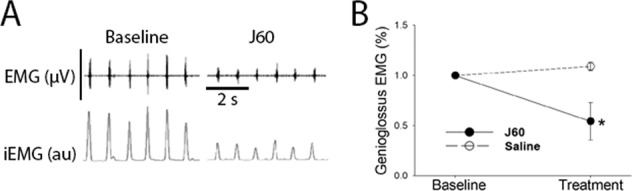
Effect of JHU37160 dihydrochloride (J60) on genioglossal muscle activity in adult mice treated with intralingual injection of AAV9 encoding an inhibitory DREADD. Data were collected 8 weeks after intralingual injection with AAV9-HA-hM4D-mCherry. **A** Representative genioglossus muscle electromyography (EMG) activity recorded at baseline (left) and after administration of the DREADD ligand J60 (right). J60 was delivered via intraperitoneal (IP) injection at 0.1 mg/kg in 250 µl saline. The top panel shows the raw EMG recording (scale bar = 40 µV) and the bottom trace shows the integrated signal (iEMG, arbitrary units, au). **B** Average inspiratory genioglossus EMG response to a single dose of J60 or saline (*n* = 7, crossover design). Data are normalized to the peak phasic EMG amplitude at baseline. Inspiratory bursting showed a time × treatment statistical interaction (*F*_1,27_ = 46.3, *P* < 0.001). *denotes a lower value in J60 vs. saline treated, *p* < 0.001.

Figure 4 shows an example of DREADD-induced manipulation of genioglossus muscle EMG activity. These data from our research group were collected in C57BL/6J mice that had diet-induced obesity described in our recent publication [44]. At 6–8 weeks following tongue delivery of an AAV9 construct driving an inhibitory DREADD (AAV9-HA-hM4D-mCherry), intraperitoneal administration of the high potency DREADD ligand JHU37160 [84] (0.1 mg/kg) caused a marked decrease in genioglossus EMG activity. These data are consistent with the reports of retrograde targeting of XII motoneurons with AAV9 [45, 46, 53] and confirm that viral-driven expression of inhibitory DREADDs be used to reduce XII motor output following intralingual AAV delivery.

Optogenetics is another approach producing rapid advances in neuroscience [86] and has the advantage of operating with high spatial and temporal resolution [86, 87]. The first direct activation of XII motoneurons with optogenetics was recently published [88]. Transgenic mice expressing channelrhodopsin-2 (ChR2) only in cholinergic neurons (ChAT-ChR2(H134R)-EYFP) were studied in vivo under anesthesia. Photostimulation using a fiber-optic probe over the XII motor nucleus caused an immediate increase in tongue muscle EMG bursting proportional to the intensity of light stimulation. The response of the system was greater in non-REM sleep and wakefulness as compared to REM sleep [88]. Optogenetic stimulation can also effectively depolarize skeletal myofibers or motor axons, typically using ChR2. This potentially offers greater spatial and temporal precision as compared to direct electrical stimulation [89]. To our knowledge these techniques have not yet been applied to the tongue and/or XII nerve, and this represents a promising research opportunity.

Several groups have also used chemo- and optogenetic methods to stimulate excitatory or inhibitory presynaptic neural pathways to better understand the neural regulation of XII motoneuron activity [90–93]. For example, medullary A1/C1 neurons were demonstrated to have an excitatory impact on XII motoneurons using a DREADD approach. A viral construct encoding an inhibitory DREADD (AAV10-hSyn-DIO-hM4Di-mCherry) was injected to the A1/C1 region in DBH-cre mice, and subsequent administration of CNO caused a decrease in genioglossus EMG output during NREM sleep [93]. Dergacheva et al. used optogenetics to explore the role of inhibitory neurons on tongue motor control [92]. Following stereotaxic delivery of AAV1-EF1a-DIO-hChR2 to the ventral medulla of GAD2-Cre mice, light activation caused inhibition of XII motor output. Interestingly, the inhibitory impact was greater for motoneurons innervating retractor vs. protrusor muscles. In the same study, in vivo chemogenetic activation of ventral medullary inhibitory neurons decreased tongue muscle EMG output. These studies illustrate the utility of chemo- and optogenetic techniques for studying the neural control of XII motoneurons, and potentially provide other neural substrates (e.g., beyond direct XII motoneuron stimulation) for therapeutic targeting.

## Conclusion

Gene therapy targeting the XII motor system has therapeutic potential for a variety of lingual-related disorders, but this potential is largely untapped. The relative ease of access to the tongue for viral delivery facilitates targeted treatment of myopathy [45]. As the field moves forward, selectively targeting individual tongue muscles, and by retrograde viral movement, their motoneurons [44], may provide a means of optimizing tongue-related gene therapy for specific needs or conditions. On the other hand, in the case of genetic disorders, delivery of a therapeutic gene may not require such specificity, but rather targeting the entire XII motor system (e.g., protrusor and retractor muscles; intrinsic and extrinsic muscles) may be appropriate. Initial successes with chemogenetic methods for selective XII motoneuron stimulation raise new possibilities for treating OSA or other conditions [80, 81]. Optogenetics may also be useful in this regard, but little work has been done in that area. Similarly, to our knowledge neither opto- nor chemogenetics have been explored in preclinical studies aimed at dysphagia and dysarthria. For all studies of gene transfer in the XII motor system, preclinical or clinical, the biomechanical and anatomical complexity of the XII motor system, and in particular the different mechanical actions of tongue protrusor vs. retractor muscles are important considerations. The importance of tongue motor control to speaking, swallowing and breathing, and the prevalence of lingual-related disorders serve to underscore the need for further research in gene transfer to the XII motor system.

## Materials and methods

This article contains original data included to illustrate concepts related to gene delivery to the XII motor system. Experimental protocols were approved by the Institutional Animal Care and Use Committees at the University of Florida (Figs. 2, 3) or Johns Hopkins University (Fig. 4). For the Fig. 2 data, a 3-month-old male 129SVE mouse received a unilateral injection to the base of the tongue with AAV9-CMV-GFP (20 µL; titer = 1.20 × 10^13^ vg/mL). The mouse was anesthetized using inhaled isoflurane and the virus was delivered to the base of the tongue as in our prior publications [44–46, 53]. Eight weeks later, the mouse was anesthetized with 3% isoflurane and systemically perfused with 0.9% saline followed by 4% paraformaldehyde. Tissues were harvested and placed in 4% paraformaldehyde for 24 h at 4 °C before sectioning using a cryostat (10 µm). Expression of GFP was visualized using a DAB (3, 3-diaminobenzidine)-peroxidase reaction as in our prior publication [85]. The data in Fig. 2 have been replicated in four published reports [44–46, 53]. For the Fig. 3 data, a 2-month-old male Sprague-Dawley rat received a 1.5 µL injection of AAV9-CBA-GFP (titer = 1.86 × 10^13^ vg/ml) to the right XII nerve under 3% isoflurane anesthesia. Nerve injection was done using a pulled glass micropipette connected to a Hamilton syringe. The micropipette tip was inserted into the main XII nerve trunk, proximal to the bifurcation into medial and lateral branches (see Fig. 1). After 8 weeks, the animal was anesthetized and perfused as described above, and brainstem tissues were harvested and then cut (40 µm) using a cryostat. Immunochemistry was performed using an antibody against GFP (Abcam; Rabbit polyclonal to GFP; lot: GR3271077) and a fluorescent secondary antibody as previously described [85]. Direct nerve injection with AAV has been demonstrated to drive motoneuron gene expression in other motor systems [78], but to our knowledge this is the first demonstration of this method in the XII motor system. To generate the Fig. 4 data, 3–4-month-old male C57BL/6J mice received an intralingual injection of AAV9-HA-hM4D-mCherry (Gi) targeting the genioglossus muscle (7 × 10^10^ vg, 10 μl in total) using the same methods described in our recent publication [44]. Mice were housed 4 or 5 per cage, in a temperature and humidity-controlled room with a 12/12 light/dark cycle (9 a.m.–9 p.m. lights on; 9 p.m.–9 a.m. lights off) with free access to water. Mice were fed with high fat diet (TD 03584, Teklad WI, 5.4 kcal/g, 35.2% fat, 58.4% kcal from fat) ad libitum. Eight weeks later after AAV9 injection, mice were anesthetized with 2–3% inspired isoflurane and then maintained at 1–2% to keep breathing rate at 60 per min. Teflon-insulated wire hook electrodes (stainless steel, Teflon-coated, A-M Systems, Carlsborg, WA) were inserted in the genioglossus muscle at the base of the tongue; wires were sutured to the neck musculature to maintain placement. The electromyogram (EMG) signal was amplified, band-pass filtered (30–1000 Hz, alternating-current preamplifier; model P511K, Grass Instruments), and digitized at 1000 Hz (LabChart Pro 7, Version 7.2, ADInstruments, Dunedin, NZ). The EMG was rectified, and a 100 ms time constant was applied to compute the moving average (LabChart Pro 7). After baseline EMG recording, mice were treated with JHU37160 dihydrochloride (J60) (0.1 mg/kg in 250 µl saline i.p.) or vehicle (saline). A total of 7 mice were studied using a crossover design. The animals received either saline or J60 on different days (2 day interval between experiments, presentation order was arbitrarily selected). An a priori statistical power calculation was not performed, and the sample size was based on prior experience with this model [44]. The experimenter was blinded to the treatment during data collection. For quantitative analysis, the peak phasic (inspiratory) components were measured for 10 consecutive breaths at baseline and 15 min following J60 or saline injection. The EMG bursts were expressed as a percent of the average phasic inspiratory baseline output. A two way repeated measures analysis of variance was used to evaluate the response to saline or J60; values are reported as mean ± 1 standard deviation. A *p* value of < 0.05 was considered statistically significant. The use of DREADDs to manipulate XII motoneuron activity has been replicated in several prior studies [44, 80–82].
